# Uncertainty estimation- and attention-based semi-supervised models for automatically delineate clinical target volume in CBCT images of breast cancer

**DOI:** 10.1186/s13014-024-02455-0

**Published:** 2024-05-29

**Authors:** Ziyi Wang, Nannan Cao, Jiawei Sun, Heng Zhang, Sai Zhang, Jiangyi Ding, Kai Xie, Liugang Gao, Xinye Ni

**Affiliations:** 1 Department of Radiotherapy Oncology, Changzhou No. 2 People’s Hospital, Nanjing Medical University, Gehu Road 68#, Wujin District, Changzhou, 213003 Jiangsu China; 2 Jiangsu Province Engineering Research Center of Medical Physics, Changzhou, 213003 China; 3 Medical Physics Research Center, Nanjing Medical University, Changzhou, 213003 China; 4 Key Laboratory of Medical Physics in Changzhou, Changzhou, 213003 China

**Keywords:** Breast cancer, Automatic delineation, Cone-beam computed tomography, clinical target volume, Semi-supervised learning, Uncertainty estimation

## Abstract

**Objectives:**

Accurate segmentation of the clinical target volume (CTV) of CBCT images can observe the changes of CTV during patients' radiotherapy, and lay a foundation for the subsequent implementation of adaptive radiotherapy (ART). However, segmentation is challenging due to the poor quality of CBCT images and difficulty in obtaining target volumes. An uncertainty estimation- and attention-based semi-supervised model called residual convolutional block attention-uncertainty aware mean teacher (RCBA-UAMT) was proposed to delineate the CTV in cone-beam computed tomography (CBCT) images of breast cancer automatically.

**Methods:**

A total of 60 patients who undergone radiotherapy after breast-conserving surgery were enrolled in this study, which involved 60 planning CTs and 380 CBCTs. RCBA-UAMT was proposed by integrating residual and attention modules in the backbone network 3D UNet. The attention module can adjust channel and spatial weights of the extracted image features. The proposed design can train the model and segment CBCT images with a small amount of labeled data (5%, 10%, and 20%) and a large amount of unlabeled data. Four types of evaluation metrics, namely, dice similarity coefficient (DSC), Jaccard, average surface distance (ASD), and 95% Hausdorff distance (95HD), are used to assess the model segmentation performance quantitatively.

**Results:**

The proposed method achieved average DSC, Jaccard, 95HD, and ASD of 82%, 70%, 8.93, and 1.49 mm for CTV delineation on CBCT images of breast cancer, respectively. Compared with the three classical methods of mean teacher, uncertainty-aware mean-teacher and uncertainty rectified pyramid consistency, DSC and Jaccard increased by 7.89–9.33% and 14.75–16.67%, respectively, while 95HD and ASD decreased by 33.16–67.81% and 36.05–75.57%, respectively. The comparative experiment results of the labeled data with different proportions (5%, 10% and 20%) showed significant differences in the DSC, Jaccard, and 95HD evaluation indexes in the labeled data with 5% versus 10% and 5% versus 20%. Moreover, no significant differences were observed in the labeled data with 10% versus 20% among all evaluation indexes. Therefore, we can use only 10% labeled data to achieve the experimental objective.

**Conclusions:**

Using the proposed RCBA-UAMT, the CTV of breast cancer CBCT images can be delineated reliably with a small amount of labeled data. These delineated images can be used to observe the changes in CTV and lay the foundation for the follow-up implementation of ART.

## Introduction

According to the 2023 Cancer Statistics, breast cancer is the most prevalent disease in women worldwide, accounting for about 31% of all cancers in women [1]. Radiotherapy (RT) after breast-conserving surgery can significantly improve the survival rate of breast cancer patients [2]. In clinical treatment, the cone-beam computed tomography (CBCT) imaging device integrated on the linear accelerator is used to obtain CBCT images, and rigid registration of CBCT images and planning CT (PCT) images is used for patient setup correction, which has been widely used in RT [3]. During setup, the radiotherapy technician needs to compare the superimposed CT and CBCT images to observe the differences and adjust the setup. When the patient is lying on the treatment bed, fast and accurate judgment is needed. The traditional registration method is slow and does not meet the clinical needs. Some studies have pointed out that in interfractional radiotherapy with a long time span, changes in body size, setup errors, and anatomical structure of patients will affect the treatment effect and increase the probability of radiation injury [4, 5]. Adaptive radiotherapy (ART) uses the online image of the patient to make treatment decisions, re-contouring and evaluation, etc., which can automatically adjust the radiotherapy plan during the fractional treatment [6], thereby reducing the influence of interfractional radiotherapy. Performing ART can improve the accuracy of treatment and is a promising method [7], among which automatic delineation of clinical target volume (CTV) on CBCT images is an important step in ART.

Due to the intrinsic characteristics of radiotherapy after breast conserving surgery, there are some difficulties in segmentation of CTV on CBCT images of breast cancer. Firstly, CBCT images are easily affected by medical equipment and patient motion, which makes CBCT images contain a large number of artifacts and low soft tissue contrast [8, 9]. Secondly, CTV is difficult to distinguish radiologically from normal tissues, which increases the difficulty of delineation. Third, the existing deep learning methods need to be trained with a large amount of labeled data to achieve good segmentation performance, which is difficult to obtain CBCT labeled data. Finally, since CTV is delineated by estimating the degree of microscopic disease spread based on accumulated knowledge of previous treatment outcomes and histological evidence of the degree of tumor cell spread for a particular cancer, CTV contours delineated by different clinicians may vary considerably [10].

At present, CBCT image segmentation has been initially explored in areas such as lung [11, 12] and pelvic region [13–15], but due to the intrinsic complexity, there are few studies on CBCT image segmentation of breast cancer. Dai et al. [16] used CycleGAN to generate synthetic CT from CBCT of breast cancer patients, and then input the 3D U-Net segmentation network trained by PCT, so as to achieve CTV segmentation on CBCT images of breast cancer. However, Yuan et al. [17] pointed out that the similarity between synthetic images and PCT in radiomics features was quite different, and some error information may be synthesized, which still needs further study. Most of the existing deep learning-based segmentation methods rely on the training of a large number of labeled data. However, it is a time-consuming and laborious process to obtain large datasets for labeled segmentation, especially for medical image segmentation that requires clinical and medical knowledge. The semi-supervised learning (SSL) segmentation method emerging in recent years can learn additional feature information in a small amount of labeled and unlabeled data to reduce the training cost [18]. The commonly used semi-supervised segmentation methods include pseudo-label [19, 20] and consistency regularization [21–23]. Methods based on pseudo-labels assign pseudo-labels to unlabeled data; however, low-quality pseudo-labels may have higher uncertainty and may contain more noise, thus having a greater impact on the performance of the model [24]. Approaches based on consistency regularisation encourage models to produce the same predictions for input images under small perturbations at the data, feature and model levels. For example, Tarvainen et al. [25] proposed the mean teacher (MT) model, which divides the network into two parts: the student network and the teacher network. The inputs of the two networks are respectively added with independent random noise, and the purpose of using a small amount of label segmentation is achieved by training the consistency of the outputs of the two networks.

In order to avoid the potential propagation errors and internal deformation problems between the forms of the synthetic images, we directly performed automatic segmentation of the CBCT images. Inspired by the idea of consistency regularized semi-supervised segmentation, we propose the residual convolutional block attention-uncertainty aware mean teacher (RCBA-UAMT) model for the automatic segmentation of CTV in CBCT images of breast cancer. The model integrates the residual module and channel spatial attention module on the backbone network 3D UNet to improve the feature extraction ability of the framework, and introduces an uncertainty estimation strategy to assist the segmentation. In the labeled data part, CT and CBCT images were input, and the rich image information of high-quality CT was used to assist the network learning. Comprehensive evaluation of our model and existing SSL methods shows that our model has higher segmentation accuracy.

## Materials and methods

### Data acquisition

A total of 60 patients with breast cancer treated with right-side breast-conserving therapy in our hospital from February 2017 to September 2023, including 60 PCT and 380 CBCT, were collected. The CTV labels on CBCT in 52 cases were obtained by the deformable registration of CTV labels on CT images to CBCT images, and then manually refined by senior clinicians. The CT and CBCT of the same patient were only used for training or testing simultaneously, and the specific data distribution is shown in Table 1**.** Only those patients who received whole breast irradiation were included in this study; therefore, patients who received axillary or supraclavicular irradiation were excluded. All patients were female, with age ranging from 30 to 72 years. The supine position was adopted with the hands crossed over the head and fixed on the vacuum pad. The PCT images of all patients were obtained by Siemens CT (Somatom Force, Germany) with a size of 512 × 512, a spatial resolution of 0.98 mm × 0.98 mm, and a slicer thickness of 5 mm. CBCT images were acquired using the XVI system from Elekta Infinity (Elekta,Stockholm,Sweden) between 2 and 4 weeks after PCT acquisition. Compared with the standard chest M20 technology, the gantry speed was increased from 180 to 360°/min using fast chest M20 technology, and the projection frame was reduced from 720 to 360, which not only reduced the patient's scanning time and radiation dose but also reduced the image quality to a certain extent [26]. The tube voltage was 120 kV, and the current was 20 mA. The kV detector panel had a field of view of 42.5 cm × 42.5 cm, a reconstruction matrix of 410 × 410, and a pixel size of 1 mm × 1 mm. The acquired CBCT images of breast cancer had a truncation. This study was approved by the Medical Ethics Committee of our hospital (#2020KY154-01).

**Table 1:** Summary of patient characteristics

Patient characteristics	CT_labeled_	CBCT_labeled_	CBCT_unlabeled_
Age range	30–72		
Train	51	31	328
Test	9	21	
Total	60	52	328

### Contour delineation

In order to reduce the influence of subjective differences in the delineation of CTV between doctors on the network, we invited an oncologist to delineate the CTV of all the included data according to a unified standard. (1) Upper margin: the upper margin of breast tissue was referred to clinical markers and CT, and the highest level of sternoclavicular joint was observed. (2) Lower margin: refer to the lower margin of breast tissue seen by clinical markers and CT, or the level of breast folds. (3) Internal margin: the inner margin of breast tissue was referred to clinical markers and CT, and did not exceed the parasternal. (4) External: referring to clinical markers and the outer edge of breast tissue visible on CT, or referring to the contralateral breast. (5) Anterior margin: 5 mm subcutaneous (mainly including breast tissue, if the breast volume is small, 3 mm subcutaneous can be considered). (6) Posterior border: excluding ribs, intercostal muscles and pectoralis major muscles. When delineating CBCT images, we first deformable registration the CTV on CT images to CBCT images, and then the doctor compared the two images and delineated the CTV on CBCT images according to the standard to form the ground true(GT) on CBCT images.

### Proposed methodology

**Fig. 1:**
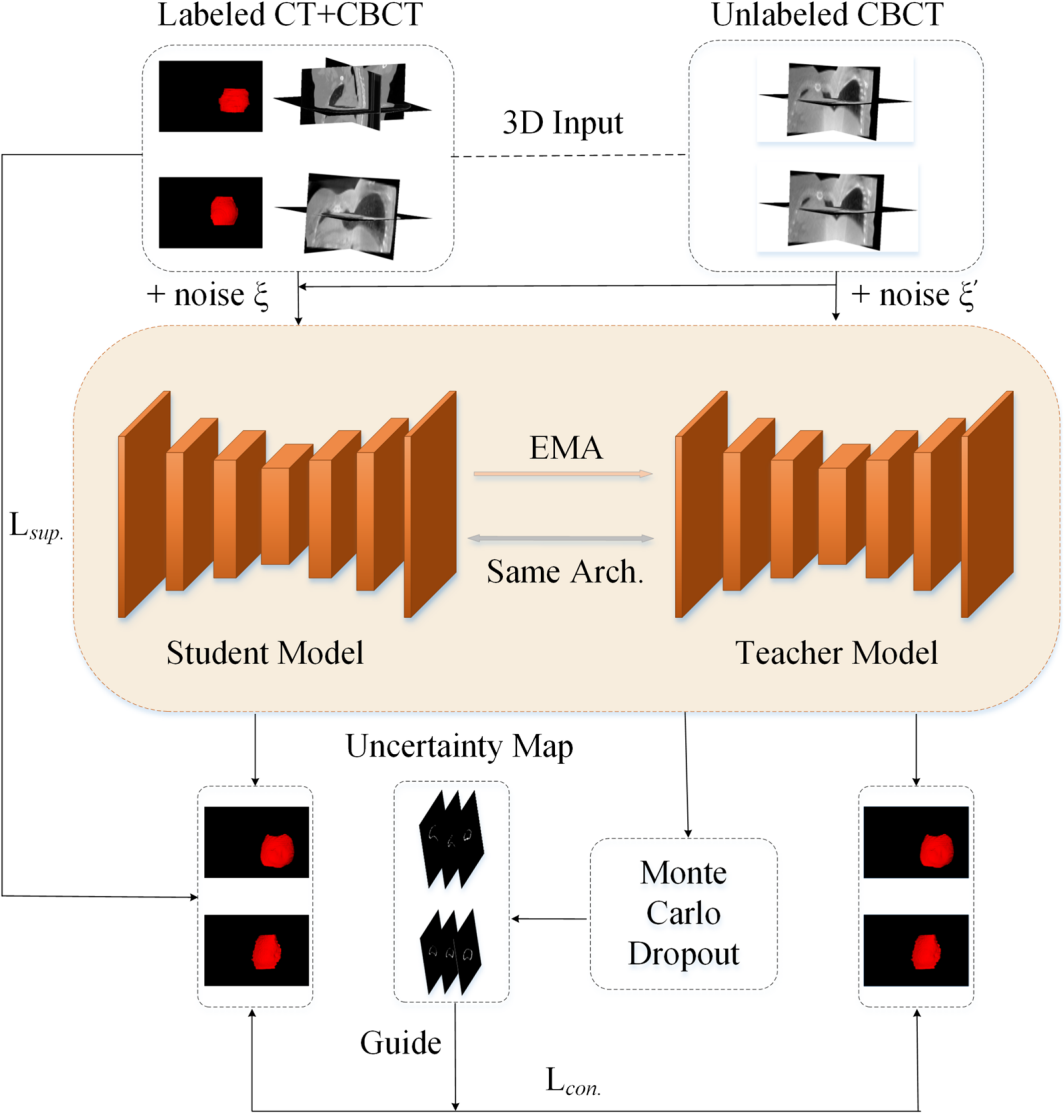
Schematic illustration of our RCBA-UAMT framework

Our proposed RCBA-UAMT is shown in Fig. 1, where labeled CT and CBCT images are inputted to the student model, and unlabeled CBCT images are inputted to the student model and the teacher model, with different noise perturbations added randomly to each input. Features are randomly lost in the teacher network, and N forward propagation is performed to obtain N sets of prediction results. Therefore, for each pixel of the input image, N groups of SoftMax probability vectors can be obtained. Subsequently, the average probability vector can be calculated. Finally, the information entropy can be calculated as the evaluation measure of uncertainty. The supervision loss, Lsup. is calculated by the student model on the input and output of the labeled image. The consistency loss Lcon. is calculated from the output of the student model and the teacher model, and utilizes the uncertainty guided consistency loss by using the information of the uncertain feature map of the target. The teacher model was optimized using an exponential moving average (EMA), which refers to the average of the student model weights. In this section, a detailed explanation of the proposed RCBA-UAMT segmentation model is given.

#### Backbone architecture

**Fig. 2:**
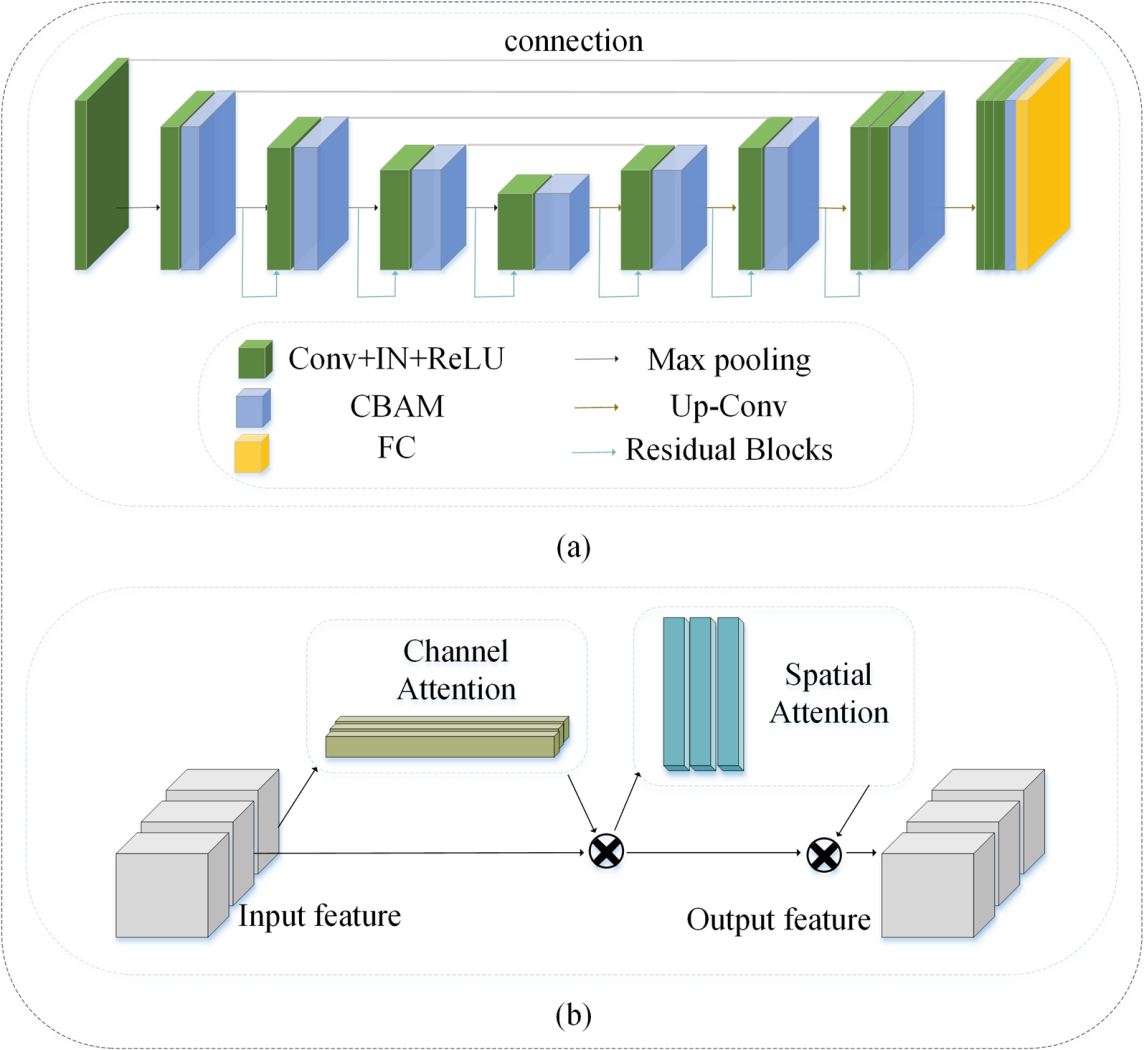
**a** Architecture of residual convolutional block attention 3D UNet, which is used as the backbone network in the RCBA-UAMT. **b** Architecture of 3D convolutional block attention module

RCBA-UAMT model with the same structure model of the teachers and students model, as shown in Fig. 2a. In this study, the residual module [27] and convolutional block attention module (CBAM) [28] are integrated on 3D UNet [29] to optimize the network. The residual module can connect the feature information between the two layers, prevent the degradation problem caused by the deepening of the network layer, and optimize the segmentation performance. As shown in Fig. 2b, the CBAM module is used to adjust the attention weight of output features from channel and space in detail to extract more effective feature information and enable the network to pay attention to more important information adaptively. In the encoder part, a convolution operation consists of a 3 × 3 × 3 convolution, InstanceNorm [30], and Leaky ReLU [31], using a maximum pooling (MaxPool) layer as downsampling. CBAM is mainly composed of two serial modules, namely, channel attention module (CAM) and spatial attention module (SAM). CAM is mainly used to perform attention weighting on the channel dimension of the input features, and the MaxPool and average pooling operations are performed after the feature map is inputted to aggregate the spatial information of the feature map. SAM is mainly used for the attention weighting of the spatial dimension of the input features. Finally, the convolution and sigmoid activation functions were used to obtain the spatial attention map, which was multiplied with the input feature map to obtain the final output feature map.

The parameters of the teacher model are obtained from the student model through EMA, and the formula is expressed as follows:

$$z_{t}{\prime} = \varepsilon z_{t - 1}{\prime} + \left( {1 - \varepsilon } \right)z_{t} ,$$

where $\text{z}$ and ${\text{z}}{{^{\prime}}}$ represent the parameters of the student network and the teacher network, respectively. $\upvarepsilon$ is the fixed value parameter, which is set to 0.99 in this study. When the teacher network is updated, 99% of its own parameters remain unchanged, and 1% is transferred from the student network.

#### Uncertainty estimation

Given that the boundary between CTV and normal tissue is fuzzy, the CTV edge is inevitably prone to uncertainty during automatic segmentation. In this paper, an uncertainty estimation method based on Monte Carlo dropout [32] is used to add uncertainty estimation to the network to provide reliable segmentation possibilities with different confidence levels and explain incorrect predictions. In this method, dropout is used to train the model so that the model parameters seem to follow a Bernoulli distribution. For each input, different outputs will be generated, and the variance of different outputs is calculated to obtain the uncertainty. Specifically, noise was added randomly to each input image and entered into the teacher network multiple times. It is used to conduct N times of forward propagation in the teacher network to obtain N groups of prediction results. Therefore, for each pixel of the input image, N groups of SoftMax probability vectors can be obtained, and the average probability vector can be calculated. The formula is expressed as follows:

$$M_{c} = \frac{1}{N}\mathop \sum \limits_{t} p_{t}^{c} .$$

The formula for calculating the uncertainty of the average probability is as follows:

$$U = - \mathop \sum \limits_{c} M_{c} \log \left( {M_{c} } \right),$$

where N is the number of forward propagation, which is set to 8 in this study; c is the segmentation category; p_t_ is the probability graph of the t degree; M is the probability map after averaging; and U is the information entropy and is the probability weighting of the entropy of all segmentation categories.

#### Loss functions

The semi-supervised 3D segmentation model was proposed to minimize the following joint objective loss functions:

$$L = argmin_{z} \mathop \prod \limits_{i = 1}^{M} L_{sup.} (f(x_{i} ;z),y_{i} ) + \lambda \mathop \prod \limits_{i = M + 1}^{M + Q} L_{con.} (f\left( {x_{i} ;z,\xi } \right),f(x_{i} ;z^{\prime},\xi{\prime} ),$$

where Lsup. represents the supervised loss function, and the Dice loss function [33] combined with the cross-entropy loss function [34] is used in this study to evaluate the segmentation quality of the labeled data. Lcon. is denoted as the unsupervised consistency loss function [35]. The segmentation neural network is denoted by $f$, $\text{z}$, and ${\text{z}}{{^{\prime}}}$, which denote the parameters of the student and teacher networks. $\upxi$ and ${\upxi }^{\prime}$ are random noises with different teacher and student models. y is the label. M is a case of labeled data. Q is a case of unlabeled data. i is the data index, and $\uplambda$ is a weighting coefficient to regulate the trade-off between unsupervised and supervised losses.

The consistency loss is only calculated in the region of low uncertainty, and the formula is expressed as follows:

$$L_{con.} \left( {f,f^{\prime}} \right) = \frac{{\mathop \sum \nolimits_{i} H(u_{i} < I)\left( {f_{i} - f_{i}{\prime} } \right)^{2} }}{{\mathop \sum \nolimits_{i} H(u_{i} < I)}},$$

where H is the sign function (u < I is 1, u > I is 0); ${f}_{i}$ and ${f}_{i}{\prime}$ are the prediction results of the student and teacher networks at the ith voxel, respectively; ${u}_{i}$ is the uncertainty of the prediction results of the teacher network; and $I$ is the uncertainty threshold used to filter the uncertain voxels.

### Construction of comparative experiments

We compare and analyze with several advanced SSL segmentation methods, including MT, uncertainty-aware mean teacher (UAMT) [21] and Uncertainty Rectified Pyramid Consistency (URPC) [36]. For fair comparison, we used the same network backbone (3D UNet) with the same epoch for testing in these methods. In addition, the above networks were trained with 5%, 10% and 20% labeled data to evaluate the effect of different proportions of data on the segmentation effect of the network. In the labeled part, the ratio of CT to CBCT data was 5:3.

Three sets of network experiments were constructed to evaluate the effects of different modules on the segmentation performance of the network. The first group is UAMT with only 3D U-Net in the backbone network, and the second group is Res-UAMT with residual fast added to the backbone network. The third group is for our proposed network RCBA-UAMT.

### Experimental setup and evaluation metrics

This study is implemented based on the PyTorch framework using the SGD optimizer to update the network parameters, the initial learning rate is set to 0.001, the batch size is 2, it consists of 1 labeled image and 1 unlabeled image, and the training epoch is 100. A sub-volume of 400 × 400 × 48 in the center of the 3D image was trimmed as the network input, and the final segmentation result was obtained using a sliding window strategy.

In this study, four indicators, namely, dice similarity coefficient (DSC), Jaccard, the average surface distance (ASD), and 95% Hausdorff distance (95HD), were used for quantitative assessment. DSC is used to measure the similarity of two sets, and Jaccard coefficient is used to calculate the problem of whether the common features among individuals are consistent and to compare the similarity and difference between finite sample sets. The larger the values of these two, the higher the sample similarity will be. ASD is used to measure the distance between two surfaces. 95HD calculates the distance between two sets and is sensitive to segmenting the boundary region. The smaller the values of these two, the higher the similarity of the two sets. DSC, Jaccard, 95HD, and ASD are defined as follows:

$$\text{DSC}=\frac{2(\text{A}\bigcap \text{B})}{\text{A}+\text{B}}$$

$$\text{Jaccard}=\frac{(\text{A}\bigcap \text{B})}{\text{A}\bigcup \text{B}}$$

$$\text{HD}(\text{A},\text{B})=\text{max}(\text{min}||\text{a}-\text{b}||),\text{a}\in \text{A},\text{b}\in \text{B}$$

$${\text{ASD}} = \frac{1}{{\left| {{\text{S}}\left( {\text{A}} \right)} \right| + \left| {{\text{S}}\left( {\text{B}} \right)} \right|}}\left( {\mathop \sum \limits_{{{\text{a}} \in {\text{S}}\left( {\text{A}} \right)}} \mathop {\min }\limits_{{{\text{b}} \in {\text{S}}\left( {\text{B}} \right)}} \left| {\left| {{\text{a}} - {\text{b}}} \right|} \right| + \mathop \sum \limits_{{{\text{b}} \in {\text{S}}\left( {\text{B}} \right)}} \mathop {\min }\limits_{{{\text{a}} \in {\text{S}}\left( {\text{A}} \right)}} \left| {\left| {{\text{b}} - {\text{a}}} \right|} \right|} \right),$$

where A represents the predicted segmentation result, B represents the GT, S (A) represents the surface voxels in the set A, and S (B) represents the surface voxels in the set B.

## Results

**Fig. 3:**
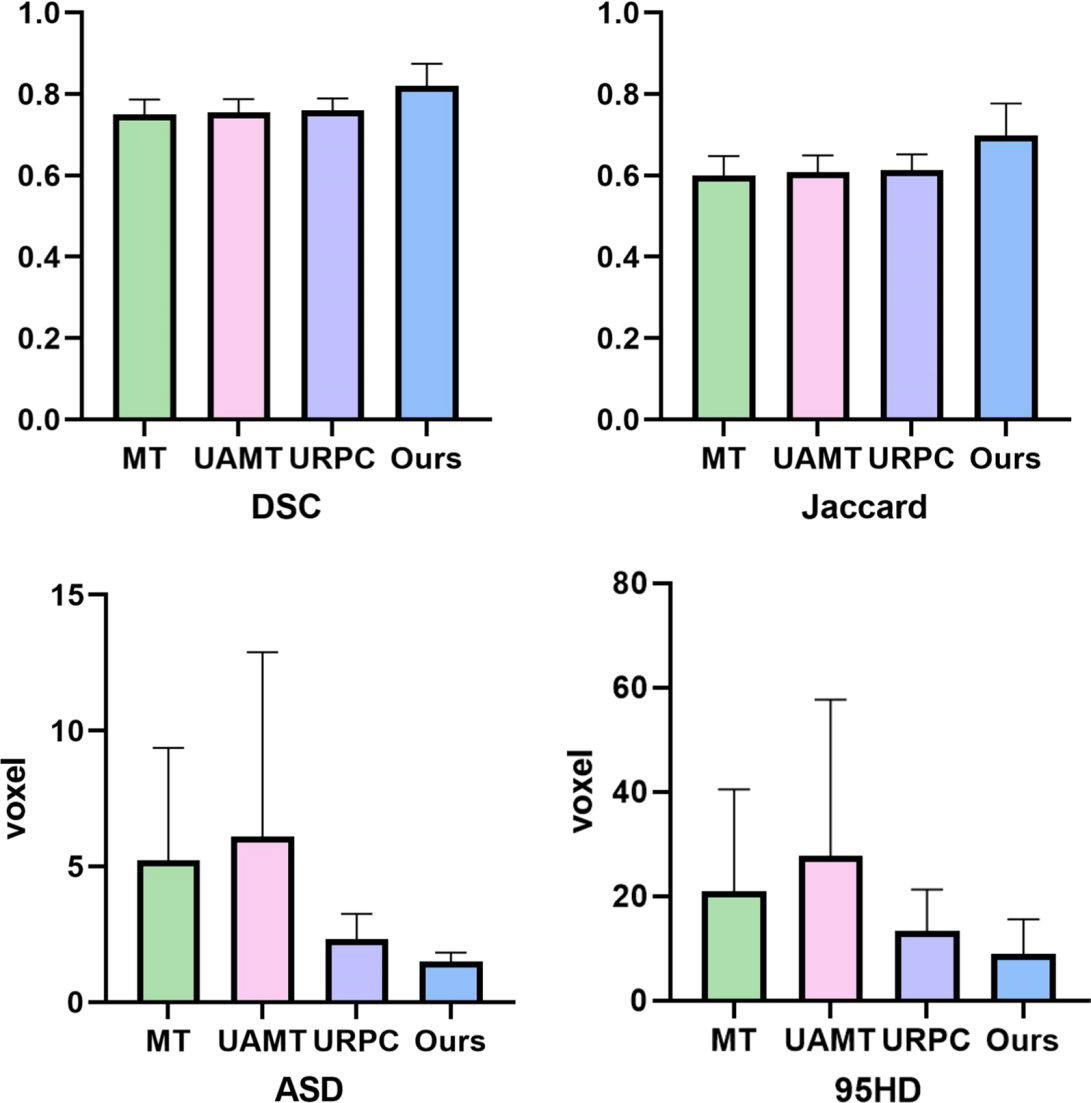
Quantitative analysis of each evaluation index of the four SSL methods was performed under 10% labeled data, with the columns representing the mean and the top representing the standard deviation

The quantitative results of labeled data with different ratios are shown in Table 2. The evaluation indexes of the method proposed in this study are better than those of several advanced SSL segmentation methods at present on 10% labeled data and 20% labeled data, especially on 95HD. Compared with the three other SSL methods on 10% labeled data, our method resulted in a 9.33%, 7.89%, and 7.89% increase in DSC, 16.67%, 14.75%, and 14.75% increase in Jaccard, 57.35%, 67.81%, and 33.16% decrease in 95HD, and 71.46% decrease in ASD, 75.57% and 36.05%. Table 3 shows whether the results obtained by using the T-test method have significant difference to calculate different proportions of labeled data. 10% and 20% labeled data have significant differences compared with 5% labeled data, whereas 10% and 20% data have no significant difference, indicating that the method proposed in this study can obtain relatively stable segmentation results on a small amount of labeled data. Thus, the cost of manual delineation is saved. Figure 3 shows the quantitative analysis plot of the four evaluation indexes, namely, MT, URPC, UAMT, and RCBA-UAMT at 10% labeled data, with the top of the cylinder as the mean value and the top as the standard deviation range, indicating that the proposed method has better effect and relatively higher stability.

**Table 2:** Quantitative comparison of the model in this paper with existing semi-supervised models

Labeled data	Method	DSC (%)↑	Jaccard (%)↑	95HD (voxel)↓	ASD (voxel)↓
21/389(5%)	MT	0.62	0.45	123.80	42.91
	UAMT	0.75	0.60	18.28	4.56
	URPC	0.75	0.60	15.55	2.78
	Ours	0.73	0.57	20.98	1.89
41/369(10%)	MT	0.75	0.60	20.94	5.22
	UAMT	0.76	0.61	27.74	6.10
	URPC	0.76	0.61	13.36	2.33
	Ours	**0.82**	**0.70**	8.93	**1.49**
82/328(20%)	MT	0.79	0.66	13.72	3.16
	UAMT	0.79	0.66	13.23	5.39
	URPC	0.78	0.64	13.21	1.75
	Ours	0.81	0.68	**8.48**	1.70

Boldface data are the best values for this column and underlined data are the second best values

**Table 3:** In our proposed method, whether the evaluation indicators results of different proportions of labeled data have significant differences

Evaluation metrics	5% vs. 10%	5% vs. 20%	10% vs. 20%
DSC	*P* < 0.05^*^	*P* < 0.05^*^	0.53
Jaccard	*P* < 0.05^*^	*P* < 0.05^*^	0.49
95HD	*P* < 0.05^*^	*P* < 0.05^*^	0.80
ASD	0.14	0.12	0.86

*Represents a significant difference

**Fig. 4:**
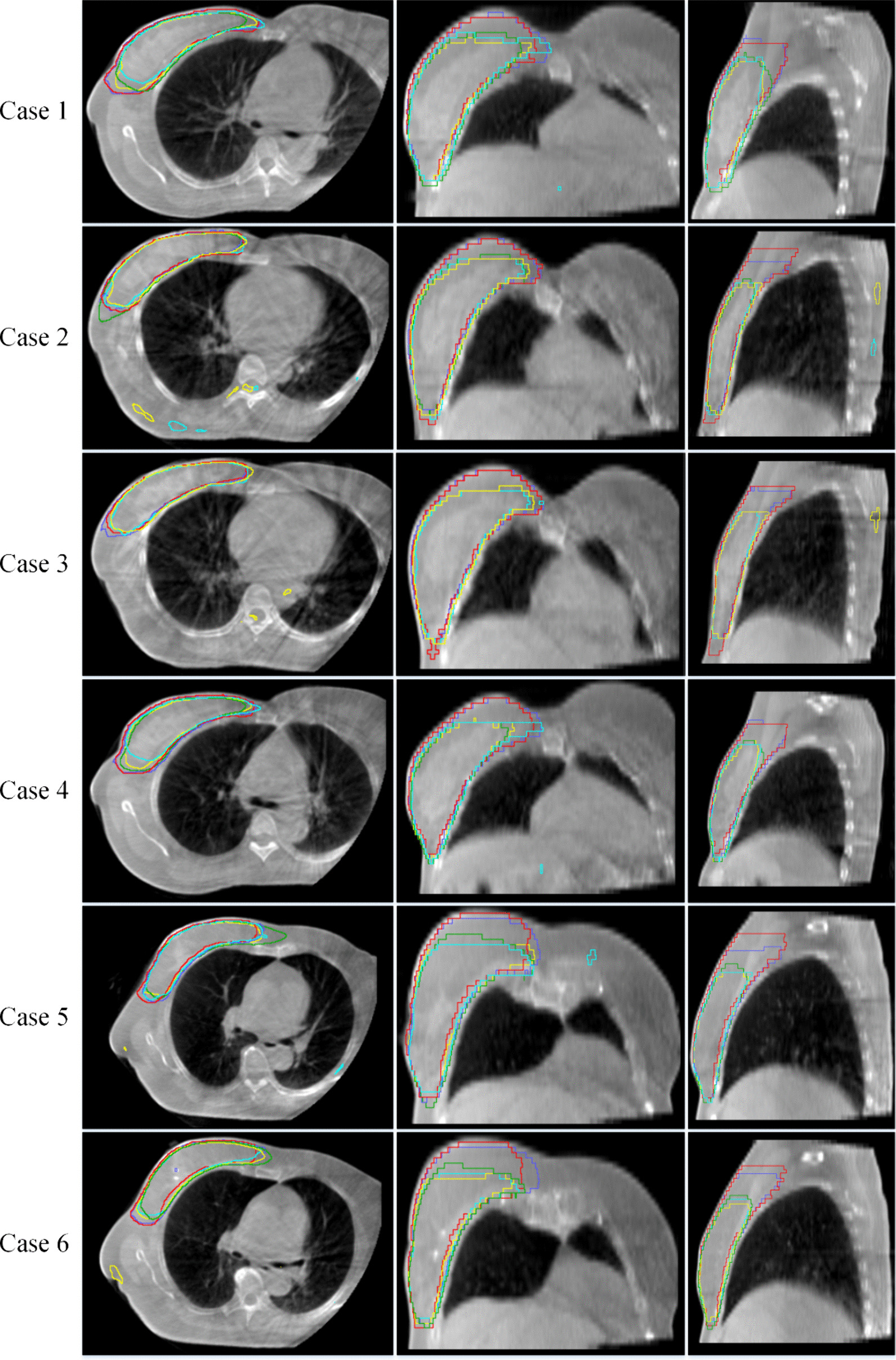
Proposed RCBA-UAMT model visual results compared with different semi-supervised segmentation models. Red represent GT, blue is MT, yellow is UAMT, green is URPC, and purple is ours method. Each row shows a different sample

Figure 4 shows the segmenting results of the four methods on CBCT when the labeled data is 10%. Red is the GT, blue is the MT segmenting result, yellow is the UAMT segmenting result, green is the URPC segmenting results, and purple is the segmenting results of the method proposed in this study. The method proposed in this study is more fully contouring and does not contour other regions. Table 4 summarizes the influence of each module on the network segmentation performance under training data with 10% labeled data. Our proposed method has remarkable performance on all metrics. Compared with the baseline model, DSC and Jaccard of the proposed model increased by 5.13% and 7.70%, respectively, whereas 95HD and ASD decreased by 35.80% and 51.94%, respectively.

**Table 4:** With 10% labeled data, comparison of proposed RCBA-UAMT with baseline model and after plus residual module in proposed model

Method	DSC (%)↑	Jaccard (%)↑	95HD (voxel)↓	ASD (voxel)↓
UAMT	0.78	0.65	13.91	3.10
Res-UAMT	0.79	0.66	11.60	2.07
RCBA-UAMT	**0.82**	**0.70**	**8.93**	**1.49**

Boldface data are the best values for this column

## Discussion

CBCT has been widely used in image-guided RT and is register with PCT to assist patient setup [37]. However, when CBCT images produce large anatomical structure changes or poor quality, the registration results with CT are poor [38], and manual correction is required, thereby increasing the time and labor cost. ART can re-optimize the planning parameters according to the anatomical changes of the patient before treatment, which requires automatic delineation of the target volume on online CBCT images. However, most of the existing CBCT segmentation focuses on the segmentation of tumor targets with normal anatomical structures and obvious differences from normal tissues, and there are few studies on CTV segmentation.

In this study, we propose the RCBA-UAMT model for automatic delineation of CTV in CBCT images of breast cancer. In order to avoid the morphological changes of the image during the synthesis process, the model was automatically contouring directly on the CBCT images. RCBA-UAMT is trained using a small amount of labeled data against a large amount of unlabeled data. Firstly, CT and CBCT images were input into the model, and the rich image information of high-quality CT was used to assist the network learning. In addition, we propose an uncertainty estimation based on MC-dropout to quantify the uncertainty by calculating the variance of each pixel of the segmentation output to obtain the information entropy on different channels, which is used to analyze the confidence of each pixel. Finally, the spatial channel attention module was integrated into the backbone network so that the model could focus on learning the segmentation information. The results showed that under the training of 10% labeled data, the average DSC, Jaccard, 95HD, and ASD of CTV delineated by our model on CBCT images were 82%, 70%, 8.93, and 1.49 mm, respectively. Our method also has several advantages. First, the direct segmentation of CBCT images can effectively avoid the deformation caused by the registration or synthesis process. In addition to bone alignment, it can be matched with the CTV label of PCT to assist radiotherapy positioning. In addition, if a large change in CTV is, then the radiotherapy plan can be adjusted in time. This study also lays the foundation for subsequent research on ART, and realized the monitoring of target dose in the whole process of radiotherapy in further research. We also believe that this method will have great potential in future clinical applications.

Our study also has some limitations. First, this study lacks a labeled dataset of all CBCT images for comparative experiments. One solution is to use a deformable registration algorithm, such as GT, to generate a large number of labels; however, this approach also introduces registration errors that are difficult to eliminate [39]. In this study, partial contouring CBCT data combined with labeled PCT were used for SSL segmentation research, and a small amount of labeled CTV data information and a large amount of unlabeled image information were learned to achieve the automatic delineation of CTV in CBCT images. In addition, this study lacks more data from open source or other hospitals for external testing and model generalization study. In the follow-up study, we will actively cooperate with other hospitals to conduct multi-institutional studies to further optimize the model and improve the segmentation performance and generalization ability of the model. Finally, automatic segmentation of organs at risk (OARs) is also an important part of ART. We will further study the content of joint segmentation of CTV and OARs in the following experiments.

## Conclusion

This study shows that the proposed RCBA-UAMT can be used to reliably delineate the CTV in CBCT images of breast cancer using a small number of labeled datasets. It can be matched with PCT label to assist patient setup, reduce setup error, observe whether the CTV changes, determine whether the treatment plan needs to be adjusted, etc., which lays a foundation for ART and target dose monitoring. The automatic delineation of CTV can reduce the burden of observation during setup and improve the consistency of delineation.

## Data Availability

The data presented in this study are available on request from the corresponding author.

## Abbreviations

CTV: Clinical target volume
RCBA-UAMT: Residual convolutional block attention-uncertainty aware mean teacher
CBCT: Cone-beam computed tomography
DSC: Dice similarity coefficient
ASD: Average surface distance
95HD: 95% Hausdorff distance
RT: Radiation therapy
PCT: Planning CT
ART: Adaptive radiotherapy
SSL: Semi-supervised learning
MT: Mean teacher
GT: Ground true
EMA: Exponential moving average
CBAM: Convolutional block attention module
MaxPool: Maximum pooling
CAM: Channel attention module
SAM: Spatial attention module
UAMT: Uncertainty-aware mean teacher
URPC: Uncertainty rectified pyramid consistency
OARs: Organs at risk
